# Glioneuronal tumor with neuropil-like islands in the spinal cord: A case report and literature review

**DOI:** 10.1097/MD.0000000000029237

**Published:** 2022-05-20

**Authors:** Honglei Liu, Can Wang, Lei Lou, Yuehong Li, Li Yi

**Affiliations:** a Department of Neurosurgery, Shijiazhuang Third Hospital, Shijiazhuang, China; b Department of Pathology, Second Hospital of Hebei Medical University, Shijiazhuang, China.

**Keywords:** glioneuronal tumor with neuropil-like islands, glioneuronal tumor, spinal cord

## Abstract

**Rationale:**

Glioneuronal tumor with neuropil-like islands (GTNI) is a distinctive neoplasm located in the cerebrum. Moreover, spinal GTNI is extremely rare. Herein, we present a case of spinal GTNI and review the related literature.

**Patient concerns:**

A 38-year-old Chinese woman presented to our hospital with a 6-month history of neck pain and a 1-month history of dizziness.

**Diagnoses:**

Magnetic resonance imaging revealed a large intramedullary mass spanning the length of the spinal cord from C1 to C4. Microscopic and immunohistochemical examinations of the tumor tissue revealed findings typical of GTNI.

**Interventions:**

The patient underwent C1 to C4 intraspinal gross tumor resection.

**Outcomes:**

Follow-up results showed that the patient had no recurrence 6 months after tumor resection.

**Lessons:**

GTNI in the spinal cord is a highly rare neoplasm with poor prognosis. Therefore, clinicians and pathologists should differentiate GTNI from other benign glioneuronal tumors, and long-term follow-up of patients with spinal GTNI is necessary.

## 1 Introduction

Glioneuronal tumor with neuropil-like islands (GTNI) is a distinctive neoplasm that was first reported by Teo et al in 1999.^[1]^ It has been reported to be located in the cerebrum and is currently considered a variant of astrocytoma, World Health Organization (WHO) grade II or III.^[2]^ Spinal GTNI is extremely rare. Herein, we present a case of spinal GTNI and review the related literature. The clinicopathological features, molecular characteristics, differential diagnosis, and prognosis of spinal GTNI were analyzed and summarized.

## 2 Case report

**Figure 1:**
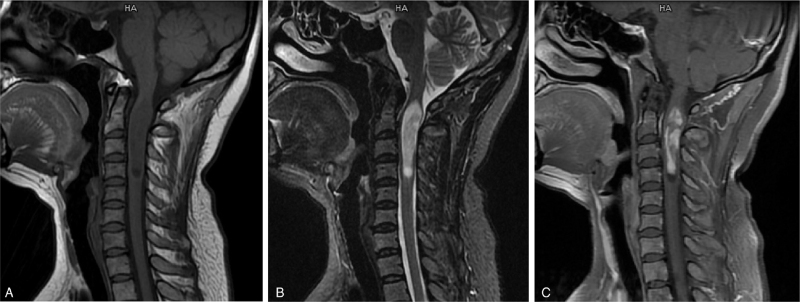
Magnetic resonance imaging reveals the primary intramedullary lesion extending from C1 to C4, characterized by T1-weighted imaging hypointensity (A) and T2-weighted imaging hyperintensity (B), and intensity is enhanced after contrast medium administration (C).

A 38-year-old woman presented to our hospital with neck pain and dizziness. She developed neck pain 6 months ago, and this symptom gradually worsened. She started experiencing dizziness approximately 1 month ago, but no neurological changes or red flag symptoms, such as weakness, sensory changes, or bowel/bladder dysfunction, were observed upon presentation. The patient's symptoms were not relieved through conservative treatment, such as oral medications and infusions (details are unknown). Therefore, magnetic resonance imaging (MRI) was performed, which revealed a large intramedullary solid mass spanning the length of the spinal cord from C1 to C4. The mass was hypointense on T1-weighted images and hyperintense on T2-weighted images, and an intense enhancement after contrast administration was observed (Fig. 1A-C). Thus, the patient underwent C1 to C4 intraspinal gross tumor resection and bone graft fusion internal fixation.

**Figure 2:**
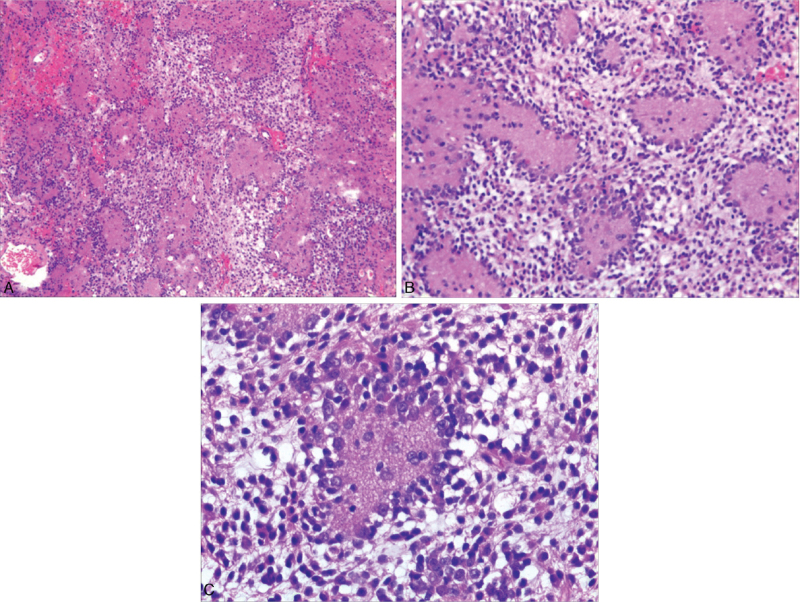
(A, B) Loose sheets of uniform, small tumor cells intervening between neuropil-like islands (hematoxylin and eosin, 40× [A]; 100× [B]). (C) Rimming an island, an astrocytic component with ganglion-like cells is observed (hematoxylin and eosin, 200×).

Macroscopically, the tumor was described as an ash-red soft mass of 2 cm × 1 cm × 1 cm. Histological examination of routine hematoxylin-and-eosin-stained sections showed scattered, differently sized, and well-defined neuropil-like islands against the background of astrocytic components (Fig. 2A, B). The neuropil-like islands comprised the outer layers of monotonous round cells and a central neuropil-like matrix. Ganglion-like cells were present and scattered within and around the neuropil-like matrix, with abundant cytoplasm and large nuclei (Fig. 2C). The astrocytic component of the tumor displayed diffuse astrocytomas with mucinous degeneration, but with no mitosis or necrosis. Therefore, it was classified as a WHO grade II tumor.

**Figure 3:**
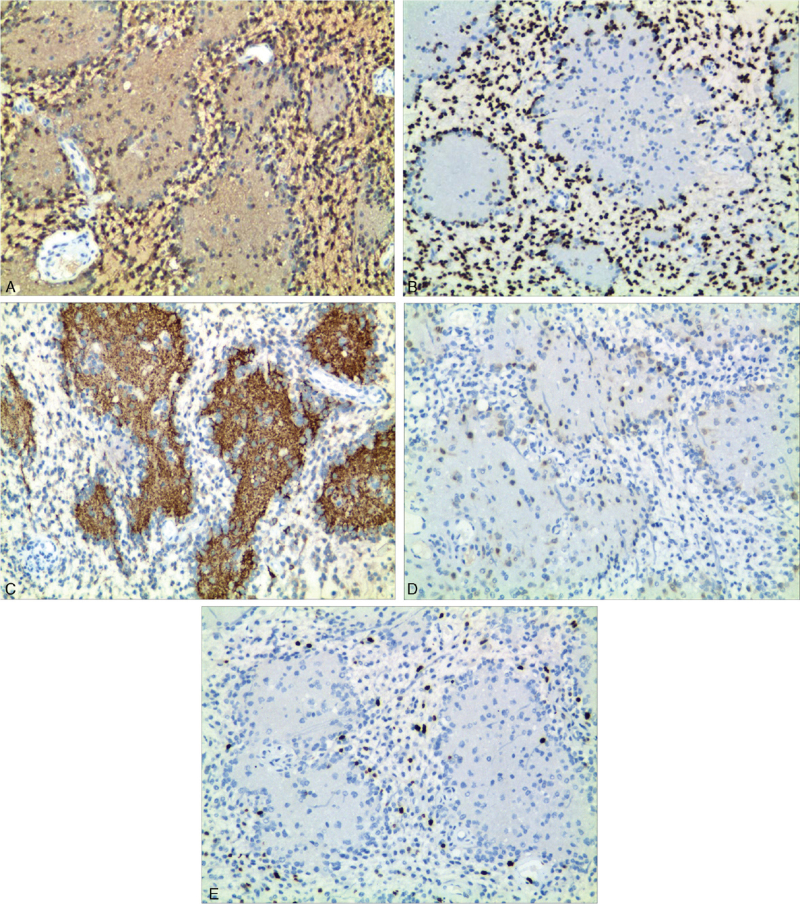
(A) Glial fibrillary acidic protein (GFAP) is strongly positive in the astrocytic component (immunohistochemistry [IHC], 100×). (B) The astrocytic component is decorated by Oligo-2 (IHC, 100×). (C) Synaptophysin immunostaining highlights the neuropil-like islands (IHC, 100×). (D) The ganglion-like cells are immunopositive for neuronal nuclear protein (IHC, 100×). (E) The Ki-67 proliferation index reached 5% in the astrocytic component and less than 1% in the neuropil-like islands (IHC, 100×).

Immunohistochemistry showed that the astrocytic component was positive for glial fibrillary acidic protein (GFAP) (Fig. 3A), oligodendrocyte transcription factor 2 (Fig. 3B), vimentin, and S-100 but negative for isocitrate dehydrogenase 1 (IDH1), H3K27M, P53, and α-thalassemia X-linked intellectual disability (ATRX). The neuropil-like material showed synaptophysin (Fig. 3C) and nestin staining, whereas the ganglion-like cells were immunopositive for neuronal nuclear protein (Fig. 3D). Moreover, GFAP staining was negative. The proliferation index determined by the proportion of Ki-67-positive cells in the astrocytic component was 5%, whereas it was <1% in the neuropil-like islands (Fig. 3E). BRAF AV600E and IDH R132H gene mutation analyses yielded negative results.

The patient's symptoms disappeared after surgery, but the coordination of the upper limbs was observed to be poor. The patient has been closely followed up for 6 months, and no recurrence has been observed on radiologic images. Although the coordination of the upper limbs has improved through rehabilitation training, it still has not reached normal.

## 3 Discussion

GTNI is considered a variant of astrocytoma in the 2007 WHO Classification of Tumors of the Nervous System, grade II or III, as its molecular profile and aggressive behavior are similar to those of infiltrative astrocytomas.^[2]^ Most cases of GTNI reported in the literature are located in the cerebrum. Primary location within the spinal cord is extremely rare, and only 12 cases have been reported to date^[3–11]^ (Table 1).

**Table 1:** Summary of all published cases of spinal glioneuronal tumor with neuropil-like islands.

	Sex	Age	Location	Clinical presentation	Disseminated	Rescue management	Molecular genetic	Grade	Accompanying symptoms	Follow-up (mo)
Harris et al^[3]^	Female	44	C7-T1	Numbness and tingling of the right fingers, weakness of the right arm, paresthesias and weakness of the right leg	Yes	Cervicothoracic laminectomy, PTR, RT, CR	ND	IV	Extensive syrinx of the cervical and thoracic cord	15 died
Ruppert et al^[4]^	Female	54	T7-T10	Intermittent numbness of the left leg progressing to bilateral, lower extremity sensory loss	Yes, leptomeningeal dissemination involving the entire craniospinal axis	T6-T10 laminectomy for tumor biopsy and debulking, RT	ND	III	Syrinx extended enhancement along the central canal	12 unable to walk
Buccolier et al^[5]^	Female	6	T11-L1	Left lower-extremity hypotonia and paretic march, multiple lesions at the posterior fossa level	Uncertain	Microsurgical partial resection of the lesion through a one-level laminectomy, RT, CT	Deletion of 1p;	III	ND	24
Buccolier et al^[5]^	Female	8	C6-T3	Back pain	ND	Laminectomy, GTR, RT	Deletion of 1p;	II to III	ND	14 recurrence
Fraum et al^[6]^	Male	48	T8-T9	Fourteen months after diagnosed with a WHO grade II oligodendroglioma with the emergence of progressive paresthesias along the entire plantar surface of the right foot and the left big toe	No	Thoracic laminectomy and open biopsy, RT, CT	1p/19q-deleted		ND	10 minor sensory deficits below T8
Serra et al^[7]^	Female	2	T12-L2	Headaches associated with intermittent vomiting due to a tetraventricular hydrocephalus	Yes	GTR, RT,CT	ND	II	ND	30
Comunoglu et al^[8]^	Male	14	C5-T1	Scoliosis and urinary incontinence	ND	GTR	ND	II	ND	
Giunti et al^[9]^	Female	6	ND	ND	Yes	PTR, CT-ACST, craniospinal RT	ND	III	ND	76 PR
Giunti et al^[9]^	Female	8	ND	ND	No	PTR, CT-ACST, focal RT	ND	III	ND	70 PR
Duan et al^[10]^	Male	47	T1-T6	Chest and back discomfort, low back pain, numbness of the right lower limb	No	GTR	1p/19q, PTEN, EGFR-	III	ND	14
Duan et al^[10]^	Male	23	C7-T3	Chest and back pain, aggravated with upper limb pain	No	Almost GTR	1p/19q, PTEN, EGFR-	II to III	ND	42 recurrence
Meng et al^[11]^	Female	46	T4-T8	Hypalgesia and athalposis in the left lower extremity, a sharp pain in the proximal part of the left lower extremity and lumbar region, dizziness, and nausea	ND	PTR	ND	III	Hydrosyringomyelia within the spinal cord from T1 to T12	ND
Present case	Female	38	C1-C4	Neck pain, dizziness	No	GTR, bone graft fusion internal fixation	BRAFV600E-IDH R132H-	II	No	6

ACST = autologous stem cell transplantation, CR = complete response, CT = chemotherapy, GTR = gross total removal, HDCT = high-dose chemotherapy, ND = not described, PR = partial response, PTR = partial total removal, RT = radiotherapy.

According to the reported cases, including the present case, the mean episode age of patients with GTNI was 26.5 (range 2-54) years, with a female predominance (9 females and 4 males). Notably, 5 of the 13 spinal GTNI cases were observed in the pediatric age group. Thus, it can be inferred that spinal GTNI is predominantly observed in young individuals. The neuroimaging characteristics of spinal GTNI have been consistently described as a large intramedullary mass, with MRI findings showing a solid mass with or without cystic components. Hydrosyringomyelia was also observed in 3 out of 13 cases.^[3,4,11]^ Although it has been reported that hydrosyringomyelia has a good prognosis, this prognostic significance does not seem to apply to GTNI. In the literature review, there were 4 cases in which the tumor originated from the thoracic vertebra, 4 in which the tumor originated from the cervicothoracic region, and 2 in which the tumor originated from the thoracolumbar region. In the present case, GTNI solely originated from the cervical vertebra. The most frequent clinical symptoms of spinal GTNI are numbness, weakness, and limb paresthesia. Other symptoms include neck, chest, back, and lumbar pain. Comunoglu et al^[8]^ presented the case of a patient who returned for follow-up due to scoliosis and urinary incontinence. Fraum et al^[6]^ have reported a case of spinal GTNI after a medical history of WHO grade II oligodendroglioma. In the present case, the patient developed neck pain and dizziness, but no neurological changes were observed.

All tumors showed a mixed glioneuronal tumor, comprising an astrocytic component and neuropil-like islands. Within and around these islands, round oligodendrocyte-like cells and ganglion-like cells were observed. Astrocytic components are usually astrocytomas or oligodendrogliomas. Other tumors of cerebral GTNI have been reported in the literature, including ependymomas^[12]^ and glioblastomas^[13]^; however, these tumors were not observed in spinal GTNI. Interestingly, neither vascular proliferation nor necrosis was observed in all spinal cases that were present in cerebral GTNI.^[5]^

The overall immunohistochemical features of this case were consistent with those of previously reported cases; the neuropil-like islands demonstrated immunoreactivity with neuronal markers, such as synaptophysin, whereas the astrocytic component displayed strong immunoreactivity for GFAP protein. Neuronal nuclear-labeled cells and a few ganglion-like cells were observed around the edges of the neuropil-like islands. In the astrocytic component, the Ki-67 proliferation index ranged from 1% to 18%. The tumor was classified as WHO grade II or III, according to the astrocytic component. In the present case, we also assessed IDH1, H3K27M, P53, and ATRX, which are typical of aggressive gliomas; however, all were negative.

Due to the extreme rarity of GTNI, there are limited reports investigating their molecular characteristics. Jason et al^[14]^ have found that every sample set (12/12) exhibited evidence of the IDH1 R132H mutation in their study. Kakkar et al^[15]^ demonstrated that p53 and ATRX mutations were observed in all 4 cases in their study and that IDH1 was positive in 3 cases. However, 1p/19q co-deletion was absent, indicating that the molecular pathogenesis of these tumors is similar to that of diffuse astrocytic tumors. Fraum et al^[6]^ have suggested that there is a genetic association between GTNI and oligodendroglioma since the patient in their case was diagnosed with spinal GTNI 14 months following a diagnosis of WHO grade II oligodendroglioma. Fluorescence in situ hybridization (FISH) analysis revealed that this lesion exhibited the same 1p/19q deletion present in the concurrent cerebral oligodendroglioma. Two of the 3 tumors had a 1p deletion in Buccoliero's study; however, 19q deletion, MGMT gene promoter methylation, epidermal growth factor receptor (EGFR) amplification, and EGFR, IDH1, IDH2, and TP53 gene mutation analyses yielded negative results.^[5]^ Duan et al^[10]^ have assessed the status of EGFR, 1p/19q, and PTEN by FISH analysis in cases of GTNI, but the results were negative. In the present case, spinal GTNI was also negative for IDH1 mutation. Furthermore, we tested BRAF V600E, a marker that can be mutated in ganglioglioma; unfortunately, the results were negative. Giunti et al^[9]^ have considered that GTNI is not a genetically homogeneous entity, as chromosome imbalances were present only in 2 of the 4 cases in their study. However, due to the inconsistency of these results, a larger number of cases need to be assessed to identify unifying features.

GTNI arising from the spinal cord needs to be differentiated from other tumors, such as rosette-forming glioneuronal tumors, intramedullary astrocytoma, and ependymomas. Among these tumors, rosette-forming glioneuronal tumor is similar to GTNI in its characteristic pathological changes, and it is considered a benign tumor featuring neurocytes involved in the formation of minute neuropil rosettes and has an astrocytic component similar to pilocytic astrocytoma. Intramedullary astrocytoma is especially hard to differentiate from GTNI in imaging findings. MRI often showed invasive tumor growth in the spinal cord and generally presented mild-to-moderate heterogeneous enhancement with unclear boundaries.^[11]^ Ependymomas were often located in the center of the spinal cord with rare eccentric growth, which could become cystic and hemorrhagic.^[11]^

According to literature reviews, the prognosis of spinal GTNIs is poor. Clinical treatment includes tumor resection combined with radiotherapy and chemotherapy. Of the 13 reported patients, the follow-up data of the 2 patients was unavailable.^[8,11]^ The follow-up duration of the other 11 patients with spinal GTNI ranged from 5 months to 76 months, with 6 cases of death, recurrence, or dissemination. One patient died 15 months after treatment and showed meningeal dissemination involving the lumbar dura and possibly the cauda equina.^[3]^ Another patient was unable to walk 10 months after leptomeningeal dissemination involving the entire craniospinal axis.^[4]^ Two patients with spinal GTNI experienced dissemination at 30 and 76 months, respectively.^[7,9]^ Two other patients experienced recurrence 14 and 42 months after surgery, respectively, and developed malignant progression from WHO grade II to III.^[5,10]^ The follow-up duration in the present case was short (6 months), and the patient presented with uncoordinated movement of the upper limbs after surgery. Although it improved through rehabilitation training during the follow-up period, it still failed to reach the normal level. Therefore, long-term follow-up is required.

## 4 Conclusion

In summary, GTNI in the spinal cord is rare and seems to be more aggressive; therefore, it is crucial to differentiate GTNI from other benign glioneuronal tumors. In addition, the pathophysiologies and biological characteristics of spinal GTNI are not yet fully understood, and long-term clinical follow-up is necessary in this regard.

## Author contributions

All authors contributed to the final preparation of the manuscript.

**Conceptualization:** Li Yi.

**Data curation and Investigation:** Can Wang, Lei Lou, Yuehong Li.

**Supervision:** Honglei Liu, Li Yi.

**Writing** – **original draft:** Honglei Liu, Li Yi.

**Writing** – **review & editing:** Li Yi.
